# Endocardite infectieuse mitro-aortique compliquée de perforation valvulaire, d’anévrisme mycotique et d’infarctus spleno-rénal

**DOI:** 10.11604/pamj.2019.32.157.13009

**Published:** 2019-04-08

**Authors:** Mohammed Bachrif, Houssam Laachach, Ilham Benahmed, Mehdi Berrajaa, Alaa Fliti, Mustapha Aziouaz, Nabila Ismaili, Noha Elouafi

**Affiliations:** 1 Service de Cardiologie, Faculté de Médecine, Université Mohammed Premier, Oujda, Maroc

**Keywords:** Endocardite infectieuses, complications, perforation de valve, Infectious endocarditis, complications, valve perforation

## Abstract

L'endocardite infectieuse est considérée comme une pathologie potentiellement grave malgré tous les progrès en diagnostic et traitement. Les valves du cœur gauche sont plus touchées et les évènements emboliques, les anévrismes mycotiques, les abcès ainsi que les perforations des valves en sont des complications redoutables. Nous rapportons le cas d'une endocardite ayant atteint les valves aortique et mitrale et qui s'est compliquée d'infarctus splénique et rénale, d'anévrisme mycotique cérébral et d'une perforation de la grande valve mitrale. L'intérêt du cas est souligné suite à la bonne évolution au décours d'un traitement médico-chirurgical en dépit de la multitude des complications.

## Abstract (en)

Infectious endocarditis is regarded as a potentially serious pathology despite the advances in diagnostic and treatment options. Valves on the left side of the heart are most affected and embolic events, mycotic aneurysms, abscesses as well as valve perforations are life-threatening consequences of this disease. We here report a case of endocarditis with aortic and mitral valve involvement complicated by splenic and renal infarction, cerebral mycotic aneurysm and mitral valve perforation. The interest in the subject of this case study is the positive medico-surgical treatment outcome obtained despite a multitude of complications.

## Introduction

L'endocardite infectieuse est une affection dotée d'une lourde morbi-mortalité, ses complications aggravent davantage le pronostic. En plus d'un traitement médical bien conduit, la chirurgie est indiquée suite à des complications septiques ou hémodynamique ou même mécaniques.

## Patient et observation

**Figure 1:**
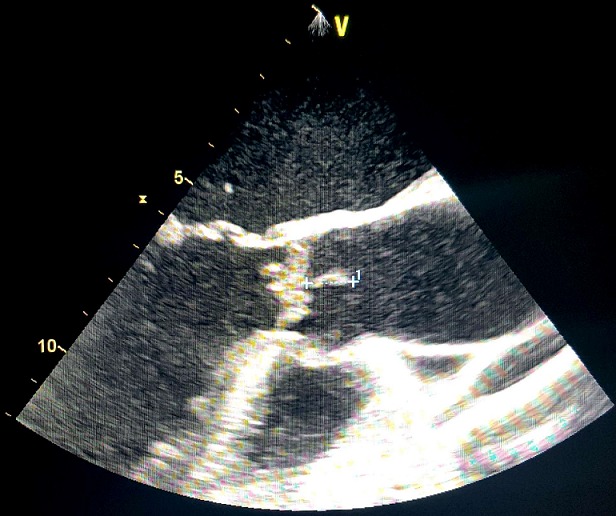
Image échocardiographique montrant une végétation aortique

**Figure 2:**
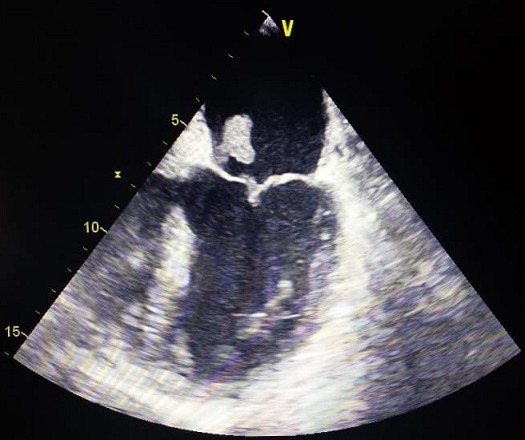
Image échocardiographique objectivant une végétation au dépend de la GVM

**Figure 3:**
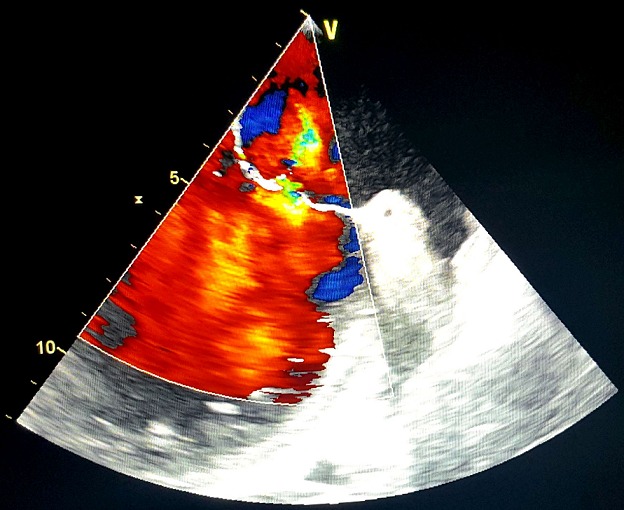
Image échocardiographique montrant l’IM et la perforation de la GVM

**Figure 4:**
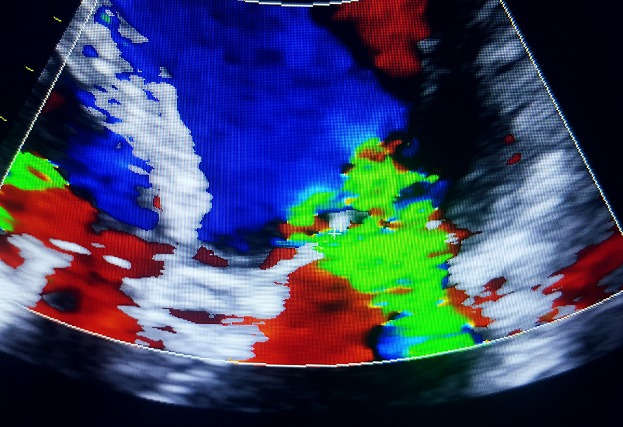
Coupe TDM cérébrale objectivant un anevrisme mycotique de l’artère cerebrale moyenne

**Figure 5:**
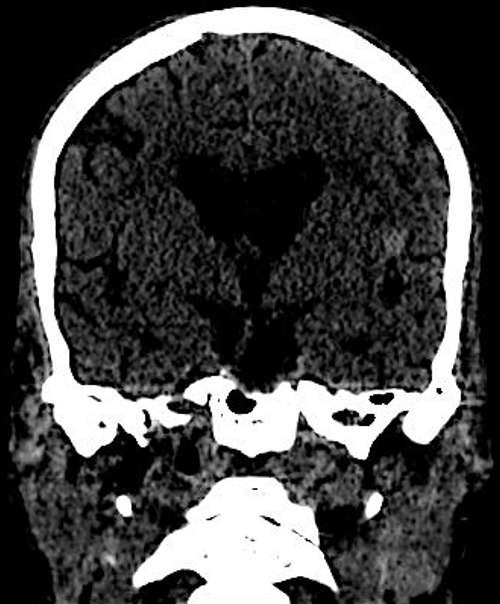
Coupe TDM montrant de petits infarcissement rénales et spléniques

**Figure 6:**
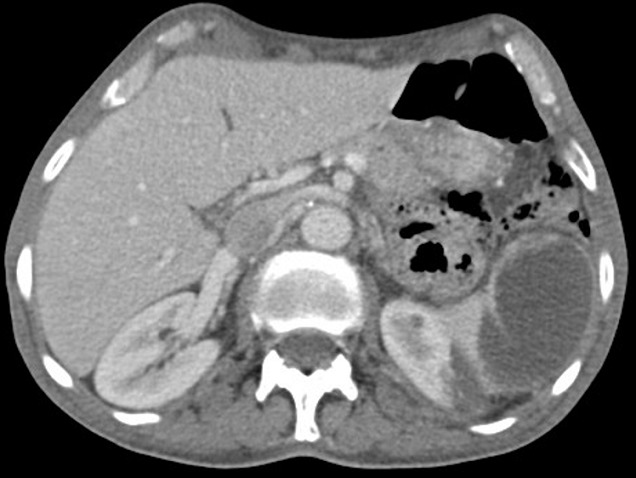
Coupe TDM montrant de petits infarcissement rénales et spléniques

Un homme âgé de 65ans, sans antécédents pathologiques notables admis pour une fièvre chronique évoluant depuis 01mois associée à une toux sèche, l'examen physique trouvait un patient conscient, fébrile à 38.3 C, stable sur le plan hémodynamique, un souffle d'insuffisance mitrale avec des œdèmes des membres inférieurs blancs mous prenant le godet arrivant jusqu'au mi-jambe, une échocardiographie transthoracique ((ETT) couplée à une échocardiographie trans-oesophagienne) étaient réalisées montrant deux végétations mobiles sur les sigmoïdes aortiques (Figure 1), une végétation mobile sur une grande valve mitrale perforée avec insuffisance mitrale importante (Figure 2, Figure 3, Figure 4), une fuite aortique modéré, un bon ventricule gauche et une hypertension pulmonaire modérée. Le bilan inflammatoire était perturbé, les hémocultures ont isolé à deux séries un enterocoque. Le bilan d'extension basé essentiellement sur une tomodensitométrie (TDM) cérébral et thoraco-abdomino-pelvien ayant montré un anévrysme mycotique cérébral (Figure 5), deux foyers d'infarcissement splénique et un petit foyer d'infarcissement rénale gauche (Figure 6). Le fond d'œil a objectivé la présence d'une tache de Roth. Le diagnostic d'une endocardite sur double valvulopathie mitro-aortique multi-compliquée a été retenu, le traitement initial était basé sur une bi-antibiothérapie initialement avec bonne évolution clinico-biologique. Une ETT de contrôle a objectivé la persistance d'une végétation de 0,5cm sur une fuite aortique modérée et la persistance d'une végétation de 0,4 × 0,9cm sur la grande valve mitrale qui est perforée avec IM importante, le patient a été traité ensuite pour double remplacement valvulaire mitro-aortique avec bonne évolution post-opératoire initiale et disparition de l'anévrisme cérébral.

## Discussion

L'endocardite infectieuse (EI) est une pathologie grave, caractérisée par une morbi-mortalité considérable du fait de ses complications [1]. Malgré tous les progrès réalisés dans le domaine de la prise en charge des EI, le nombre de malades ayant présenté une complication ou plus et ayant nécessité une prise en charge chirurgical est resté inchangé au fil des années [2]. L'entérocoque, comme isolé chez notre patient, n'est pas le germe le plus fréquemment isolé dans les endocardites simples ni celles compliquées; il acquière en effet la troisième place [1, 2]. Dans plusieurs études européennes, l'accident vasculaire cérébrale (AVC) ischémique constitue 20 à 60% des complications neurologiques de l'endocardite infectieuse surtout au niveau du territoire de l'artère cérébrale moyenne [3]. L'incidence réelle des événements emboliques est inconnue, avec des estimations allant de 10 à 50% des EI [4]. Les embolies cérébrales sont parfois inaugurales et associées au pronostic le plus mauvais avec une mortalité de 21 à 81 % [4, 5]. Les embolies coronaires sont également de très mauvais pronostic mais exceptionnelles. Les embolies spléniques, rénales et certaines embolies cérébrales sont fréquemment totalement asymptomatiques et découvertes par des examens paracliniques systématiques lors de la recherche de complications à distance [6]. Pourtant l'infarcissement rénal peut engendrer une insuffisance rénale aigue. Les perforations de valves cardiaques sont redoutables et peuvent rapidement être mortelles en dehors d'une chirurgie urgente. Leur fréquence reste mal précisée, leur diagnostic est posé par l'échocardiographie trans-thoracique et trans-oesophagienne comme chez notre patient; l'échocardiographie 3D permet une visualisation meilleure de la perforation [7].

L'anévrisme mycotique complique 2,5 à 10% des cas d'endocardite infectieuse, il peut se développer au dépend de nombreuses artères: l'aorte, les artères cérébrales, viscérales et périphériques avec rarement une localisation multiple [1, 8]. Leur risque de rupture est important (entre 38 et 50%) avec un taux élevé de mortalité (entre 40 et 60%) [9]. L'angioscanner est l'examen le plus utile pour objectiver les anévrysmes aortiques infectieux, ainsi que ceux cérébraux, possédant en outre l'avantage d'être plus disponible que l'angio-IRM (imagerie par résonance magnétique) [8, 9]. En imagerie, ils sont caractérisés par leur croissance rapide, une absence de calcification de la coque, leur aspect multilobé, leur forme sacculaire et une infiltration des tissus mous. l'anévrisme mycotique cérébral peut parfaitement disparaitre sous une correcte antibiothérapie [7-9]. L'endocardite multi-compliquée reste de très mauvais pronostic, avec une mortalité assez élevée par retentissement septique et hémodynamique. En plus d'une antibiothérapie efficace, la chirurgie s'impose le plus souvent dans les plus brefs délais pour écarter la mise en jeu du pronostic vital. Notre patient est un exemple des cas ayant bien évolué malgré la sommation de plusieurs complications.

## Conclusion

La gravité du pronostic de l'endocardite infectieuse s'alourdie par l'addition des complications type d'anévrismes mycosiques, d'événements emboliques septique, d'abcès et de perforations valvulaire ou pariétales. L'attitude thérapeutique fait appel le plus souvent à la chirurgie en association à une antibiothérapie bien menée comme en cas de perforation valvulaire ou d'autres complications auxquelles le traitement médical s'avère insuffisant.

## Conflits d’intérêts

Les auteurs ne déclarent aucun conflit d'intérêts.
